# Predicting iatrogenic adrenal insufficiency in neonates exposed to prolonged steroid courses: do cortisol levels help?

**DOI:** 10.1038/s41372-024-01996-2

**Published:** 2024-05-20

**Authors:** Kristen Rosano, Saya Bery, Jaime Marasch, Ryan Farrell, Lucy D. Mastrandrea, Rita M. Ryan

**Affiliations:** 1 Rainbow Babies and Children’s Hospital, Case Western Reserve University, Cleveland, OH USA; 2 Jacobs School of Medicine and Biomedical Sciences, University of Buffalo, Buffalo, NY USA

**Keywords:** Predictive markers, Endocrine system and metabolic diseases, Endocrine system and metabolic diseases

## Abstract

**Objective:**

To determine whether random cortisol levels obtained in neonates to assess for secondary adrenal insufficiency (AI) after prolonged steroid exposure are predictive of central AI.

**Study design:**

Data were collected on neonates born 2017–2022 who received ≥10 consecutive days of systemic steroids and had cortisol measured thereafter. Data were then collected on whether those neonates developed signs of AI or had a failed adrenocorticotropic hormone (ACTH) stimulation test.

**Results:**

Of the 71 cortisol levels (in 67 neonates) that were analyzed, there was no difference in cortisol levels between neonates who developed AI (median cortisol level of 6.5 mcg/dl) and those who did not (median of 9.2 mcg/dl), or between those who failed their ACTH stimulation test or passed it, using Wilcoxon ranked sum tests.

**Conclusion:**

These findings demonstrate that cortisol levels may not be helpful in identifying AI in neonates exposed to prolonged steroids.

## Background

**Fig. 1:**
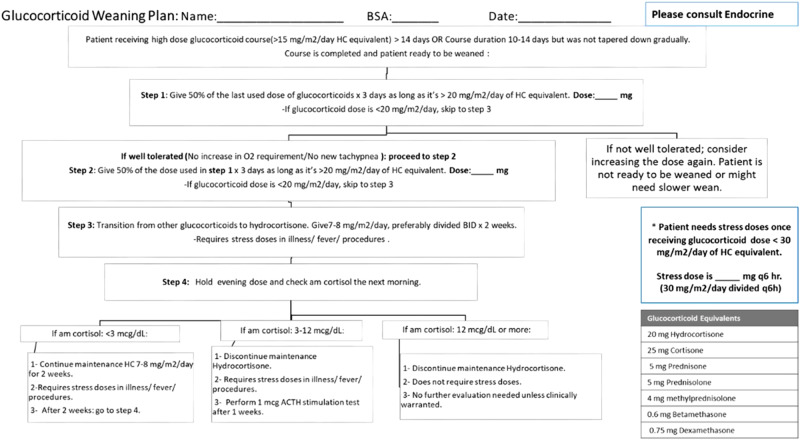
Steroid weaning protocol. Previous unit protocol for weaning steroids in neonates, which utilizes random cortisol levels to determine if steroids can be safely stopped.

Steroids are often used in premature neonates to improve respiratory status in order to facilitate extubation or wean respiratory support. Prolonged glucocorticoid exposure can cause secondary adrenal insufficiency (AI) due to suppression of the hypothalamic-pituitary-adrenal axis. Recovery of the axis depends on the total steroid dose, potency, and length of glucocorticoid exposure [1]. Our current NICU protocol (Fig. 1) for evaluating when steroids can be safely stopped involves weaning steroids by 50% every 72 h to a physiologic dose (hydrocortisone 7–8 mg/m^2^/day divided q12h) for 2 weeks. At the end of 2 weeks, the evening dose is held, and a morning cortisol level is done; that level is used to determine whether steroids can be stopped, or whether the baby needs a low-dose adrenocorticotropic hormone (ACTH) stimulation test to further evaluate for central AI. There is limited evidence in the literature that morning or random cortisol levels are predictive of central AI in this population [2, 3]. However, when this protocol was developed some years ago, this strategy was felt to be the optimal approach.

Cortisol is secreted by children and adults in a pulsatile pattern every 15–30 min in a circadian rhythm with peak cortisol levels in the early morning. Neonates do not develop this circadian rhythm until 2–3 months after birth [4, 5]. In addition, neonates in the intensive care setting may not establish their circadian rhythm until much later. Two studies have shown that peak cortisol levels in neonates occur in the evening, as opposed to the normal morning peak in adults [5, 6]. Cortisol levels have also been shown to decrease with postnatal and postconceptional age (when measured between 1 and 8 weeks postnatal age), making it difficult to interpret cortisol status in the preterm infant [7].

There have been several studies evaluating suppression of cortisol levels obtained after steroid exposure in neonates [7, 8], but no study has been done to evaluate the predictive value of cortisol levels for AI when these levels are obtained after a course of glucocorticoid treatment. The objective of this study was to evaluate whether cortisol levels obtained per our previous NICU protocol in neonates after a prolonged (≥10 days) steroid course are associated with central AI, as determined by either (1) clinical symptoms of adrenal insufficiency that required treatment, (2) the need to restart steroids due to signs of adrenal insufficiency, or (3) a failed ACTH stimulation test. Our hypothesis is that random cortisol levels do not predict central adrenal insufficiency due to the variability of cortisol levels at this age, and the lack of a circadian rhythm in neonates. Data from this study will be used to revise our clinical protocol for preterm infants requiring glucocorticoids, with the goal of decreasing lab draws and tests, hospital costs, and unnecessary steroid exposure.

## Methods

A retrospective chart review was conducted on neonates born between 2017–2022 (when the AI protocol was in place) who received at least 10 days of systemic glucocorticoids and who also had a cortisol level measured after the steroid course. For each steroid course, data were collected on whether the neonate developed concerns for possible AI, as defined as (1) the patient developing clinical concerns for possible AI (hypotension, hypoglycemia, or hyponatremia) that caused the clinical team to intervene for one of these signs (e.g., a glucose bolus), as described in the patient chart, within 7 days of stopping steroids; (2) the clinical team restarting steroids within 7 days due to concerns for possible AI; or (3) the patient failing a low-dose ACTH stimulation test within 30 days of the enrolling cortisol level. This retrospective study was approved by the Institutional Review Board at University Hospitals, Cleveland, OH (#STUDY20220579).

An initial sample size calculation determined that 80 total cortisol levels (8 linked to AI and 72 without AI) would be required to not miss a difference of 5 µg/dl in cortisol levels associated with/without AI with a power of 80% and an *α* of 0.05. This was assuming a 10% incidence of AI in our population (an estimate), and a standard deviation of 4.7 for cortisol levels, calculated using the limited literature available [9, 10].

**Fig. 2:**
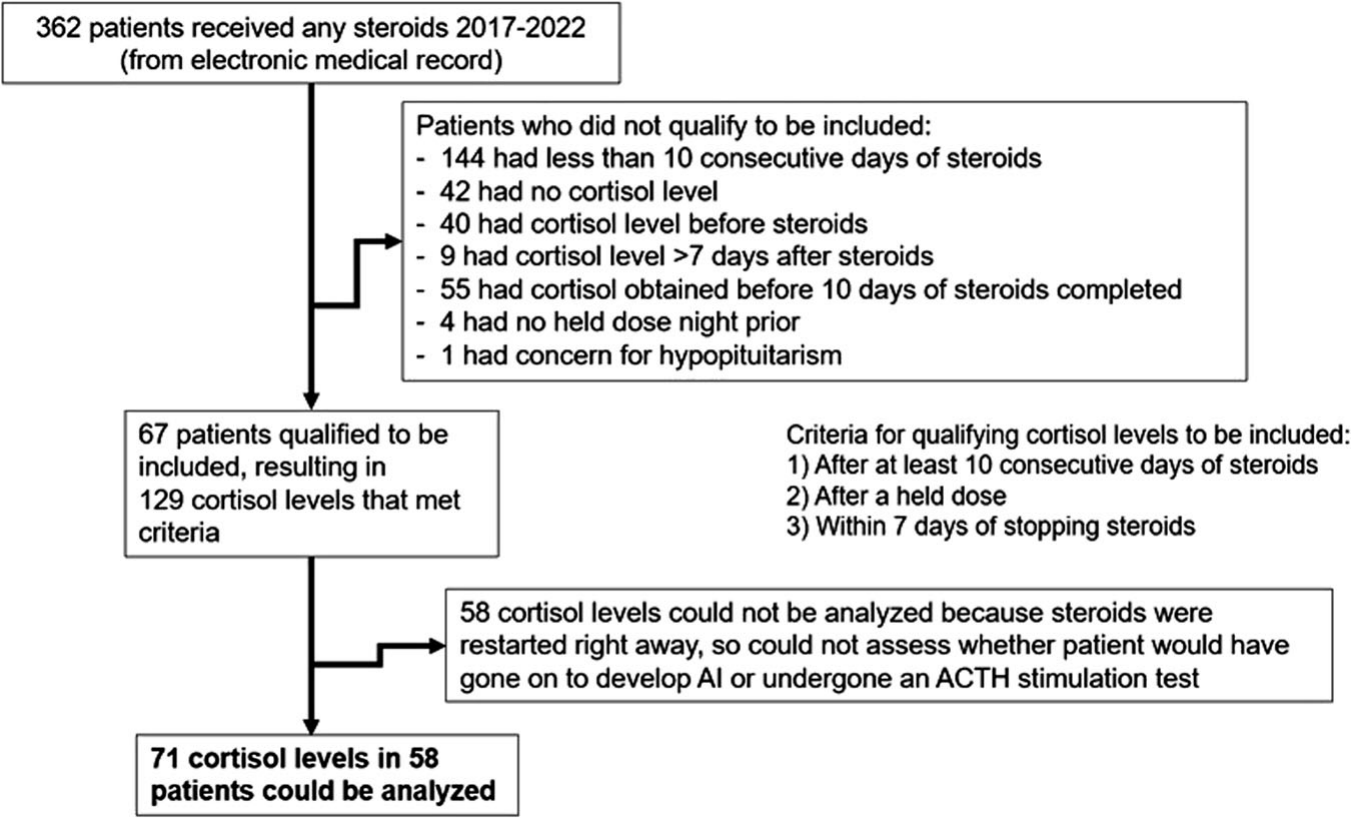
Cortisol levels included in analysis. Breakdown of steroid courses prescribed during 2017–2022 in the NICU, and cortisol levels that qualified to be included in analysis.

In the specified time period while the previous protocol was in place, 71 cortisol levels obtained in 67 neonates met criteria to be included in the analysis (Fig. 2). Some neonates had multiple steroid courses and thus multiple cortisol levels; all eligible levels were included. To be included, the cortisol levels had to be measured after at least 10 days of consecutive steroids (with at least part of that course being >15 mg/m^2^/day of hydrocortisone equivalent), after at least one held steroid dose, and within 7 days of stopping steroids. Of the cortisol levels that qualified, 58 could not be analyzed because steroids were restarted immediately after obtaining the level (protocol recommended resuming steroids if level <3 mcg/dl), so it was not possible to determine whether these patients would have gone on to develop clinical AI. Cortisol levels were compared between patients who had signs of AI and those who did not. Wilcoxon rank sum tests were used since the cortisol levels were not normally distributed, and analysis was performed using STATA statistical software (version 15, Stata Corp, College Station Texas). A *p* value of <0.05 was considered significant.

## Results

Of this population of patients with at least 10 days of consecutive steroids and a qualifying cortisol level, the median days of exposure was 70 days (Table 1), in this high-risk population. This cohort had a median gestational age of 25 weeks and birth weight 700 grams, typical of premature infants who receive steroid courses.

**Table 1:** Characteristics of patients and steroid courses.

Patient characteristics (*n* = 67)	
	Median (25th %ile, 75th %ile)
Gestational age at birth (weeks)	25 (24, 27)
Birth weight (grams)	700 (610, 888)
Characteristics of steroid exposure for each patient (*n* = 67)	
Day of life at start of first steroid course	17 (5, 29.5)
Postmenstrual age at start of first steroid course (weeks)	28 (26, 31)
Postmenstrual age at first cortisol level (weeks)	41 (34, 39)
Total number of days of steroid exposure	70 (40, 136)
Total cumulative steroid dose (in mg/m^2^ hydrocortisone equivalent)	2115 (620, 4430)
Days of extra steroids received due to low cortisol level or levels	25 (13, 43)

We found an overall incidence of clinical AI related to 6 out of 71 cortisol levels (8.5%) (Table 2). Only two of 36 ACTH stimulation tests (5.6%) performed resulted in a failed test. Analyses showed no difference in cortisol levels between neonates who developed signs of AI or failed an ACTH stimulation test, and those who did not. In addition, of the 27 neonates who at some point had a low cortisol level or levels (<3 µg/dl), 24 automatically received additional steroids per the protocol, resulting in a median of 25 extra days (interquartile range 13–43 days) of steroid exposure.

**Table 2:** Comparison of protocol-mandated cortisol levels (µg/dl) in patients with or without concerns for adrenal insufficiency.

Did patient have the following concerns for AI?	Cortisol levels (Median (IQR))	
	Yes	No
1. Signs of AI^a^ that required clinical team intervention	6.5 (3.1, 12.7) (*n* = 4)	9.2 (4.8, 15.3) (*n* = 67)
2. Clinical team restarted steroids due to signs of AI	8.7 (2.9, 14.4) (*n* = 2)	9.0 (4.8, 15.3) (*n* = 69)
3. Signs of AI based on either clinical team intervention or restarting steroids	6.5 (2.9, 14.4) (*n* = 6)	9.2 (4.8, 15.3) (*n* = 65)
	**Yes**	**No**
Failed ACTH stimulation test	10.4 (1.6, 19.2) (*n* = 2)	5.0 (2.8, 6.4) (*n* = 34)

There were no differences in levels between AI and no AI groups by Wilcoxon rank sum test.

*AI* adrenal insufficiency, *ACTH* adrenocorticotropic hormone, *IQR* interquartile range.

^a^Hypoglycemia, hyponatremia, hypotension.

**Fig. 3:**
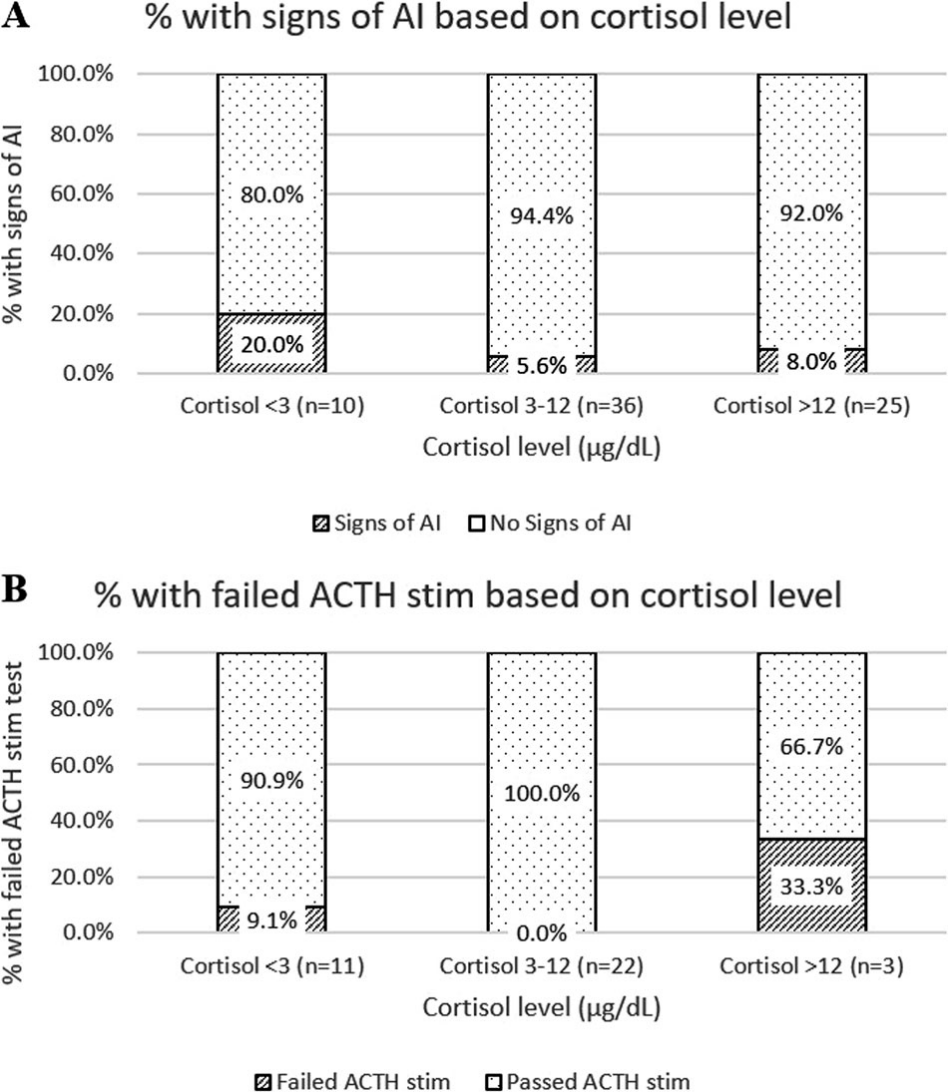
Incidence of adrenal insufficiency by cortisol level. Percent of cortisol levels that were associated with signs of AI (**A**) or a failed ACTH stimulation test (**B**) based on breakdown of cortisol levels after previous evening dose held into low (<3 µg/dl), medium (3–12 µg/dl), and high (>12 µg/dl) levels, as defined in our unit protocol.

We examined our previous protocol for its utility based on the category of cortisol level (<3 µg/dl, 3–12 µg/dl, and >12 µg/dl), (Fig. 3A, B). No differences were identified among the cortisol level groups, by Fisher exact test. In Fig. 3A, the signs of AI were defined as either having signs of AI that required intervention, or having to restart steroids due to signs of AI.

## Discussion

Random cortisol levels, obtained based on our previous protocol to determine when it is safe to stop steroids, are likely not associated with central AI in neonates exposed to prolonged steroids, as there was no difference between cortisol levels obtained in neonates who developed signs of AI and those who did not. In addition, clinical AI was rarer than expected, despite many of these patients having an ICD-10 code for AI in the medical record. Only 8.5% of cortisol levels obtained were associated with the patient having clinical signs of AI requiring intervention or for which steroids were restarted by the clinical team due to signs of AI. Only 5.6% of ACTH stimulation tests performed resulted in a failed test (peak cortisol <18 µg/dl at 30 or 60 min). The true incidence of secondary AI among all neonates exposed to steroids may be even lower than these numbers suggest, because patients with longer and repeated steroid courses had more cortisol levels obtained, so the results are skewed toward those patients with the highest steroid exposure. However, our data are also skewed because the protocol mandated restarting steroids if the cortisol level was <3 ug/dl, so possibly the most at-risk group was not given the chance to show signs of AI. Although the initial indication for starting steroids was not collected, given our typical unit practices it can be assumed that most were started and continued on steroids for prevention or treatment of bronchopulmonary dysplasia or chronic lung disease.

There have been several prior studies evaluating cortisol levels in preterm neonates, and most have also shown that cortisol levels are not predictive of adrenal insufficiency, though only two small studies looked at cortisol levels obtained after steroid exposure, as our study does. In one study of 25 premature infants, patients who received dexamethasone treatment (5 patients, for a mean of 16 days) had cortisol levels (measured at 1, 2, 4, and 8 postnatal weeks) that were not significantly different from those babies who did not receive dexamethasone, though ACTH levels were lower in infants who received dexamethasone [7]. This suggests that ACTH levels may indicate some degree of hypothalamic-pituitary axis suppression, but cortisol levels did not appear to be affected by steroid treatment. In another study of 23 very low birth weight infants who received a 3-week tapering course of dexamethasone, both ACTH and cortisol levels (obtained during a corticotropin-releasing hormone stimulation test) were significantly lower at the 3-week mark compared with prior to the steroid course. Four weeks after treatment, ACTH levels had recovered, while stimulated cortisol levels remained lower than baseline [8].

Two other studies examined cortisol levels in preterm infants, unrelated to steroid exposure. One study measured cortisol levels every 2 weeks in all neonates born <29 weeks’ gestation, and found that there was no difference in the cortisol levels obtained in neonates who later went on to develop AI versus those obtained in neonates who did not [2]. Another study looked at 11 preterm infants (with matched controls) with clinical signs of late-onset AI and measured cortisol and cortisol precursor levels before giving hydrocortisone, and found that cortisol levels did not differ between the cases and controls, but the concentration of steroid precursors was higher in the group with adrenal insufficiency, indicating that there was a limited ability to synthesize sufficient cortisol for the degree of clinical stress in those infants [3].

There are some limitations to our study. First, our initial sample size calculation determined that we would need 80 total cortisol levels (8 in babies with AI and 72 in babies without AI, assuming 10% of the cohort would have AI) to not miss a difference of 5 µg/dl in cortisol levels between AI and non-AI babies. We were only able to identify 71 random cortisol levels that qualified despite looking over a 5-year period. The steroid weaning protocol was not in place before that time, and is no longer being used, limiting the time period available. In addition, there was a lower incidence of clinical AI (8.5%) than we initially predicted (10% was used for the sample size estimate). The standard deviation of cortisol levels (7.5 µg/dl) was also higher than predicted (4.7 µg/dl, calculated as a weighted average from limited data available in the literature) [9, 10]. With this new information, the power to not miss a difference of 5 µg/dl in cortisol levels with an *α* of 0.05 was only 33%. However, based on our actual data, we had 80% power to not miss a difference of 9 µg/dl in cortisol levels between patients with and without AI.

Another limitation to this study is that for many neonates with cortisol levels <3 µg/dl, it could not be assessed whether they would go on to develop clinical signs of AI, because steroids were immediately resumed, per protocol. This could bias the results because those patients with the lowest cortisol levels presumably would be the ones most likely to develop AI, yet those patients were not followed prospectively. Thus, our estimate of clinical AI is reflective of infants with moderate (>3 mcg/dl) random cortisol levels.

Despite these limitations, our study had enough power to not miss a difference of 9 µg/dl in cortisol levels between patients who developed AI and those who did not in the population of babies with levels >3 mcg/dl, suggesting that cortisol levels may not be associated with secondary AI in neonates after prolonged steroid exposure. In addition, AI and failing an ACTH-stimulation test were uncommon (8.5% and 5.6% respectively). Based on our protocol, babies with levels <3 mcg/dl automatically resumed maintenance hydrocortisone. These neonates were likely receiving unnecessary continued steroid exposure in response to low, yet non-diagnostic, cortisol levels, and received a median of an additional 25 days of steroids due to these low levels. Based on the findings presented here, we have revised our NICU protocol with the goal of reducing unnecessary steroid treatment, blood draws, and associated cost of cortisol level testing. We have based our new approach on that of the “Buffalo” protocol, published in a review article [1] which heavily relies on observation time in hospital post-steroid course as an important factor. In general, we believe this problem can be managed without cortisol levels.

## Supplementary information

Dataset 1

## Data Availability

Dataset is available in the Supplementary Materials (de-identified and date shifted to protect identities of patients).
